# Accurate Positioning System Based on Chipless Technology

**DOI:** 10.3390/s19061341

**Published:** 2019-03-18

**Authors:** Nicolas Barbot, Etienne Perret

**Affiliations:** 1 Univ. Grenoble Alpes, Grenoble INP, LCIS, F-26000 Valence, France; etienne.perret@lcis.grenoble-inp.fr; 2 Institut Universitaire de France, 75005 Paris, France

**Keywords:** batteryless sensors, chipless sensors, wireless sensors

## Abstract

In this paper, we present an accurate method to localize an object on a 2D plan using the chipless technology. This method requires a single antenna and a chipless tag. Phase difference between a reference position and an unknown position is used to estimate the distances between each resonator and the antenna. Then, multi-lateration is used to determine the position of the chipless tag in the plan. This method provides a better accuracy compared to classical ones based on received signal strength indicator (RSSI) or round-trip time-of-flight. In a square of 10 cm side above the antenna, error over distance determination between each resonators and the antenna is less than 2 mm and localization error on the tag coordinates in the 2D plan is lower than 1 cm. To increase the robustness of this method, we propose also a selection of a subset of the resonators used by the multi-lateration process. This method permits to increase the localization area by more than 20%. All the results have been obtained in real environment, and at different heights to show the robustness of the proposed approach. Finally, localization sensors based on this method can also be used as classical chipless RFID tag for identification with the same coding capacity.

## 1. Introduction

Classical user interface requires a physical contact between the device and the user. It can be, for example, to press a button to switch on an equipment (e.g., a television), or move an equipment to point a location on a plane (e.g., a computer mouse on a screen). In each case, the user interface size depends only on the allowed interactions with the user, which introduce numerous constraints on the device size.

Contacless user interface leverages this limitation by providing a convenient solution to control an electronic device without any assumption on its size. Classical methods are based on computer vision to recognize the gesture of the user, which require direct line of sight between the camera and the user. Thus, even if the size of the equipment is not limited by the user interface, new constraints appear with the camera positioning and field of view. Moreover, since camera modules are fully passive, gesture recognition cannot be done without ambient light.

Radar based sensors, on the other side, can be used for gesture recognition irrespective of the lighting conditions. This approach can allow accurate estimation of movements and real time implementation. However, complex post-processing algorithms have to be realized to detect and classify movements from the received electro-magnetic signals. Several prototypes have already been developed [1,2]. Soli project [1] uses a Frequency Modulated Continuous Wave (FMCW) radar at 60 GHz to detect very subtle signal variations and can produce more than 10,000 range-Doppler images per second. These images are then feed to a deep convolutional neural networks to achieve features extraction and classifications of the different gestures. In [2], authors use a similar radar architecture, but process the data using a long recurrent all-convolution neural network.

The objective of this paper is to localize accurately an object on a 2D plan without any contact. Localization in 2D can be done with the previously presented solutions however, in both cases, the approaches suffer from the lack of an analytical model to determine accurately and then with low computational effort, the position of the object. Different approaches have been explored to overcome these limitations, for example, in [3] authors have developed a programmable battery-free sensing and computational platform called WISP in which data can be read by a classisal RFID reader. The WISP includes an accelerometer which can be used to determine the position of an object from the values produced by the sensor. In this paper, we proposed a new method based on the use of a chipless tag.

Chipless RFID technology has been originally design to identify items at a very low cost compared to classical RFID. However, this technology gives the opportunity to use chipless tags beyond their original desings. Indeed, the signal received by the reader carries information which is function of the tag itself (used for identification) but also information which depends on its vicinity that can be used for sensing applications. Moreover, sensing can be realized without limiting the coding capacity of the chipless tag. Sensors based on chipless tags offer contactless, low-cost, and batteryless solutions which can be deployed in real environments. Many researcher have investigated the use of chipless tags to realize sensors for temperature [4,5,6], humidity [7], gaz detection [8], level of fluids [9] and positioning [10].

In this paper, we propose a totally different approach than the ones introduced in [1,2]. Our method is based on the use of chipless tag to provide an analytical model to accurately realize the localization inside the environment and also simplify the post-processing. Since we investigate the problem of 2D localization of an object on which a chipless tag is placed, the problem is equivalent to localize the chipless tag (with the object) in the plane. Moreover, to be robust, the method should work at different heights, and should be insensitive to the other moving objects present inside the environment.

**Figure 1:**
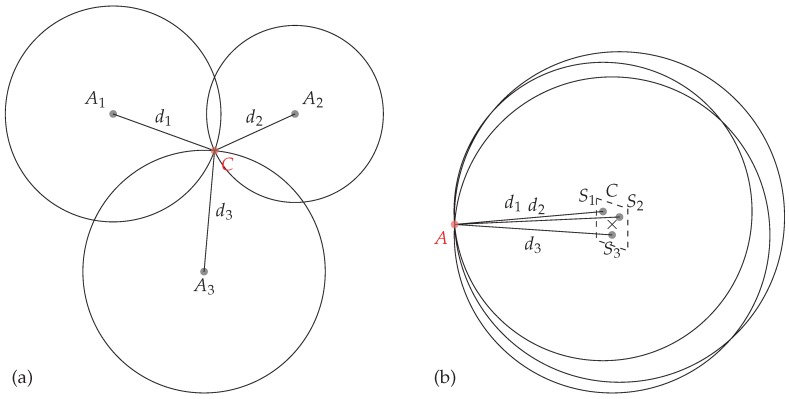
Principle of the multi-lateration used for localization (**a**) classically used in the literature, (**b**) proposed in this paper, where $A_{i}$ are the antennas, *C* the chipless tag, $S_{i}$ the scatterers. In both cases antenna positions are known, chipless tag position is unknown.

Localization based on chipless tags has already been investigated in the literature. The classical method used in [11,12,13,14] requires at least three different antennas and estimates the distance between the tag and the known antenna positions with the round-trip time-of-flight. Tri-lateration algorithm is then used to recover the position of a tag from the estimated distances. The classical setup configuration is presented in Figure 1a. All these approaches use at least 3 different antennas ($A_{i}$ in Figure 1a) and have been focused on the structural mode of the tag (i.e., the early part response of the backscattered wave) to determine the tag’s position. These approaches could easily be extended to localize the tag in the 3D space but remain sensitive to unknown objects present in the environment. In [15], the author has used the phase of the reflected signal to measure distance variations. This method provides a better robustness against the presence of unknown or moving objects in the environment since antenna mode of the response is used, however, displacements are measured along a single direction. Moreover, in [16] authors have determined the location of a chipless tag using the change of the magnitude of the backscattered signal using the information of each of the tag’s scatterer. This latter method provides an absolute positioning on a single plane but suffers from a relatively low accuracy. Finally, in [10], authors use phase measurements to localize the tag in a single plan (at a single height) and in a relatively limited area. The approach described in this paper uses the same analytical model than [10,15] but introduces a robust technique to localize the tag at different heights and over a larger area.

Our method, presented in Figure 1b, uses a single antenna *A* and relies on the phase measurement of the antenna mode of the tag to estimate the distance between the antenna and all the resonators $S_{i}$ present on the tag. Even if, tri-lateration can be used to localize a tag with only 3 resonators, classical chipless tags are composed of a greater number of resonators. Thus, this approach can easily be extended to localize the tag with a multi-lateration technique to increase the robustness of the results. Moreover, we will see that the selection of the resonators can also be made during the multi-lateration stage to increase the localization area. Our 2D localization method has been applied at different heights to highlight the performance of the approach. Finally, the presented method permits to localize the tag on a 2D plan but also does not reduce the coding capacity of the tag since localization uses the same resonators as the ones used for identification. Thus, classical reading and sensing could be combined to design new kink of sensors.

The paper is organized as follow, in Section 2, we present the RFID chipless technology and we introduce the analytical model used to determine the distance variation and multi-lateration algorithm used to localize the chipless tag. Section 3 presents the performance of our approach for distance determination and localization, at different heights in real environment. Finally, Section 4 concludes the paper.

## 2. Materials and Methods

### 2.1. Chipless RFID Technology

**Figure 2:**
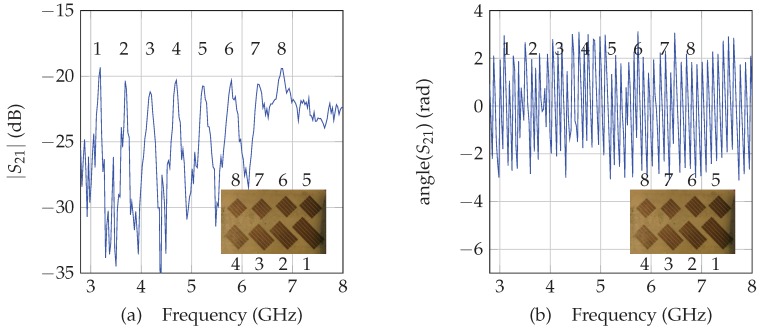
Response of the chipless tag in cross polarization [18] in (**a**) magnitude and (**b**) phase as a function of the frequency.

Chipless technology gather multiple different designs and can use different principles. Chipless tags are usually separated between time domain tags (in which the information is coded in time) and frequency domain tags (in which the information is coded in frequency). In this paper we consider a selective frequency tag since these tags provide a relative high coding capacity for a very compact size [17]. Frequency selective chipless tags are classically composed of several resonators; each resonator is a simple structure (e.g., short-circuit dipole, C-shape resonator, loop resonator...). Moreover the resonant frequency associated with each resonator is a function of a simple design parameter (typically its length). The tag ID is linked to the position in frequency of the different peaks (or deep) which compose the tag. To realize the reading of a chipless tag, the reader has to be able to generate an RF power over a large bandwidth (using an harmonic sweep or a UWB pulse) and to receive the backscatter signal as a function of the frequency to estimate the position of the peaks. Chipless tag reading is basically based on the magnitude of the received signal. Figure 2a presents the classical cross-polarization response of the tag used in this study, in magnitude. The proposed method to localize a chipless tag is based on the phase of the received signal. Figure 2b presents for the same tag, the phase of the backscattered signal as a function of the frequency.

In Figure 2a, we can clearly identify the peaks associated with each resonators. Moreover, since our localization method is based on the phase of the backscattered signal (see Figure 2b), this implies that localization can be realized without reducing the coding capacity of the tag (the same resonators can be used for reading and sensing). The next sections present the method used to determine the distance between the tag and the antenna, and to localize the chipless tag.

### 2.2. Distance Determination

**Figure 3:**
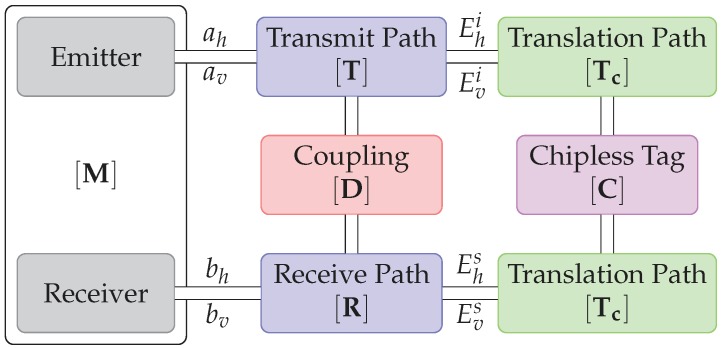
Channel model of the system for displacement measurements with one chipless tag.

The model of the transmission between the reader and the chipless tag [15] is presented in Figure 3.

The detection system is represented by the block M. Blocks T and R represent respectively the transmitting and the receiving path. Coupling between emitting and receiving antenna is noted D and block C represents the chipless tag. Since the tag can be located at a given distance *d* form the antenna, blocks $T_{c}$ represent the phase offset due to propagation and is equal to:

(1) $$T_{c}=\left[\begin{array}{cc} e^{-jkd} & 0\\ 0 & e^{-jkd} \end{array}\right]$$

where *d* is the distance between the tag and the antenna. From this model, one can extract the signal received in cross-polarization at two different positions $d_{0}$ and $d_{1}$:

(2) $$\left\{\begin{array}{cc} M_{vh}^{{}^{\prime }\left(d_{0}\right)} & =I_{vh}+T_{hh}C_{vh}e^{-j2kd_{0}}R_{vv}\\ M_{vh}^{{}^{\prime }\left(d_{1}\right)} & =I_{vh}+T_{hh}C_{vh}e^{-j2kd_{1}}R_{vv} \end{array}\right.$$

where $I_{vh}$ is the direct coupling between antennas, also called isolation measurement.

From (2), a distance variation Δd can thus be extracted by using only 2 measurements $M_{vh}^{{}^{\prime }d^{\left(0\right)}}$ and $M_{vh}^{{}^{\prime }d^{\left(1\right)}}$ at 2 different locations $d^{\left(0\right)}$ and $d^{\left(1\right)}$, and a background measurement $I_{vh}$. Distance variation $d^{\left(1\right)}-d^{\left(0\right)}$ can thus be extracted and is equal to:

(3) $$\Delta d=d^{\left(1\right)}-d^{\left(0\right)}=-\frac{1}{2k}\mathrm{angle}\;\left(\frac{M_{vh}^{{}^{\prime }d^{\left(1\right)}}-I_{vh}}{M_{vh}^{{}^{\prime }d^{\left(0\right)}}-I_{vh}}\right)$$

Note that this equation holds in cross-polarisation and only at a resonant frequency since at other frequencies, reflections from the environment could be higher than the response of the tag.

**Figure 4:**
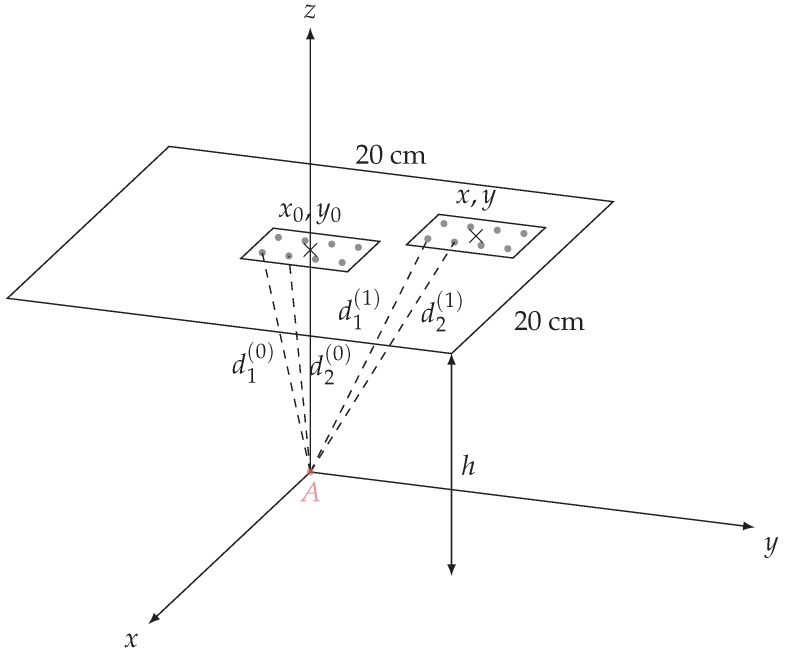
Architecture of the configuration used for localization showing the reference position $x_{0},y_{0}$ and an unknown position x,y.

An unknown distance (e.g., $d^{\left(1\right)}$) can thus be determined if the other distance is known (e.g., $d^{\left(0\right)}$) and assuming a displacement $|d^{\left(1\right)}-d^{\left(0\right)}|<\lambda$. Details can be found in Figure 4. Finally, if the tag contains more than one resonator, the distances can also be determined independently for each resonator. In the following the position in which distances are known is called the reference position.

### 2.3. Localization

As the relative positions between resonators are known (see Figure 2), and distances between resonators and the antenna can be extracted with the previously described method, it is possible to localize the tag using multi-lateration algorithms (as shown in Figure 1b) if at least three distances and the antenna position are known. Solutions based on Gauss-Newton methods have been developed in [19,20] but remain relatively complex and assume additive white gaussian noise on the estimated distances. In this paper, we consider a simpler and robust optimization process to minimize the square error between the estimated distances (obtained form measurements) and the distances obtained form a candidate solution. Details of the procedure are given in Section 3. The following section describes the measurement bench used to localize a chipless tag.

### 2.4. Measurement Bench

**Figure 5:**
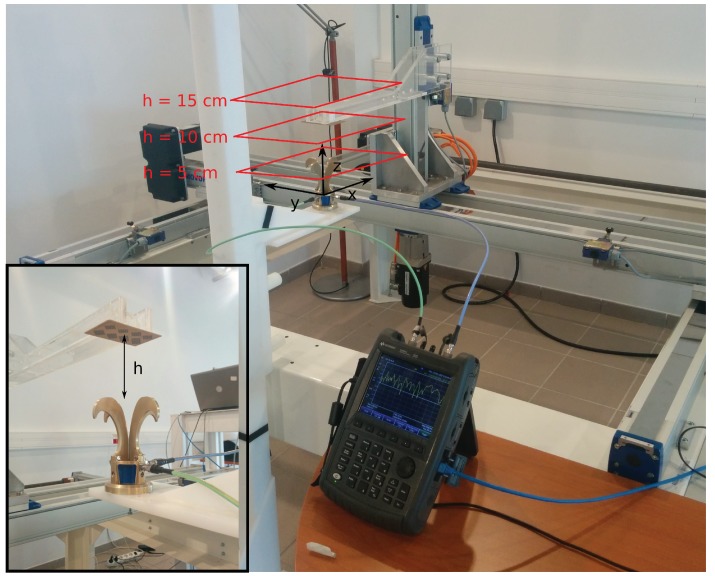
Measurement bench in real environment used for localization.

The measurement bench used in our study for chipless tag localization is presented Figure 5. The chipless tag is placed at the end of a plastic arm which can move along the three dimensions *x*, *y* and *z*. The Satimo QH2000, which is two-port antenna used to measure the signal in both vertical and horizontal polarization, is fixed and located under the tag. This antenna is connected to port 1 and 2 of the VNA. Cross-polarization response of the tag corresponds to the $S_{21}$ parameter and is acquired over the bandwidth 2.8–8 GHz. Response of the tag is measured and stored for each position in a square of 20 cm side (with a 1 cm step along *x* and *y*) above the antenna and for three different heights *h* which are 5 cm, 10 cm and 15 cm (see Figure 5). All the measurements has been realized in real environment.

## 3. Results and Discussions

### 3.1. Performance of Distance Determination

**Figure 6:**
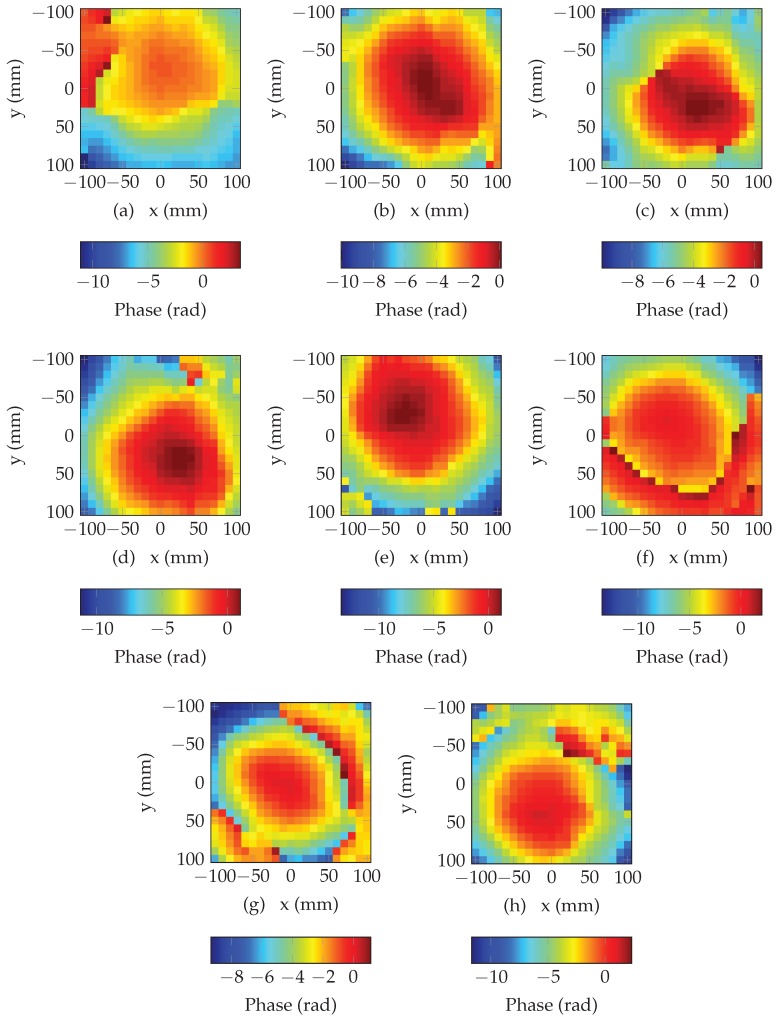
Unwrapped phase as a function of the position on the plan, (**a**) for the 1st (3.19 GHz), (**b**) for the 2nd (3.68 GHz), (**c**) for the 3td (4.18 GHz), (**d**) for the 4th (4.70 GHz), (**e**) for the 5th (5.22 GHz), (**f**) for the 6th (5.79 GHz), (**g**) for the 7th (6.33 GHz), (**h**) for the 8th resonant frequency (6.78 GHz) for the reference position $x_{0}=0$, $y_{0}=0$ and at a height of h=10 cm from the antenna.

Distances estimation $d_{i}^{\left(1\right)}$ is obtained by adding the distance of the reference position $d_{i}^{\left(0\right)}$ and the distance variation Δd obtained with (3) which uses the phase difference between the two positions. In the following, reference position has been set to $x_{0}=0$, $y_{0}=0$, and a height of h={5,10,15} cm from the antenna. Moreover, since the phase belongs to the interval [−π;+π], for displacement greater than the wavelength, phase difference is not proportional to the displacement. To estimate the correct phase value for displacement greater than the wavelength, phase need to be unwrapped. This operation consists to add multiples values of ±π to the original phase to avoid any phase shift. Even if this process can easily be applied for 1D signal, phase unwrapping for 2D signal is a complex task since modification of the phase has to be done jointly along *x* and *y*. In this work, we have used the algorithm developed in [21] to unwrap the phase in 2D. Figure 6 presents the unwrapped phase for all the resonators of the tag presented in Figure 5. We can see that the unwrapping process produces a smooth phase variation along *x* and *y*, however, for some areas, we can clearly observe important phase shift due to error during the unwrapping process (top left corner in Figure 6a and top right corner in Figure 6h) due to a low signal to noise ratio. Results produced with multi-lateration algorithm will be affected by these erroneous distances.

**Figure 7:**
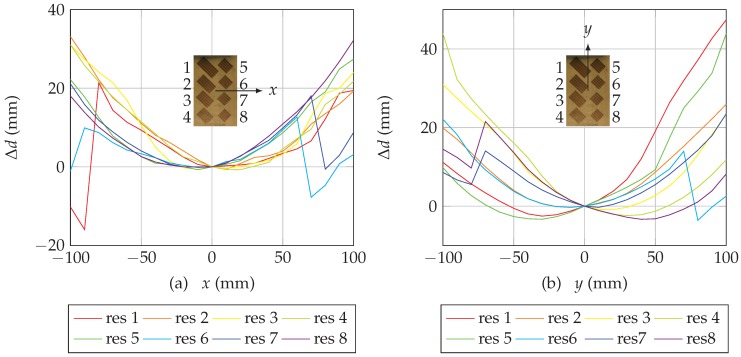
Estimation of the distance variations for the 8 resonators as a function of a displacement for a reference position located at $x_{0}=0$, $y_{0}=0$ and at a height of h=10 cm along (**a**) *x* and (**b**) *y*.

Estimation of the distance can be realized using the unwrapped phase obtained previously and (3). Figure 7 presents the evolution of the distance for all the resonators when the chipless tag is moved along *x* and *y*. We can note that all curves intersect at (0,0) since this position has been choosen as the reference position $x_{0}$, $y_{0}$ (where phase difference is equal to 0). Moreover, in Figure 7a, from the reference position, we can see that when *x* increases (tag moving to the right), distances for resonators 5, 6, 7 and 8 and the antenna increase, for resonators 1, 2, 3 and 4, distances decrease and then increase since these resonators are located on the other side of reference position. Same observations can be extracted from Figure 7b. Finally, for some resonators, (resonators 1, 6 and 7 in Figure 7a and resonators 6, 7 and 8 in Figure 7b), we can observe the effect of unwrapping errors since distances undergo sudden changes of 1 wavelength. Since λ is shorter for high resonant frequency, the unwrapping error value is smaller for these resonators (but can appear more often for the same displacements).

### 3.2. Performance of Localization Determination

Multi-lateration can be used to determine the position on the 2D plan from the distances $d_{i}$ between the antenna and each resonators. The objective of the multi-lateration is to solve a system of eight equations:

(4) $$\left\{\begin{array}{c} \hat{d_{1}}=\sqrt{{\left(x+x_{1}-x_{A}\right)}^{2}+{\left(y+y_{1}-y_{A}\right)}^{2}+{\left(z+z_{1}-z_{A}\right)}^{2}}\\ \hat{d_{2}}=\sqrt{{\left(x+x_{2}-x_{A}\right)}^{2}+{\left(y+y_{2}-y_{A}\right)}^{2}+{\left(z+z_{2}-z_{A}\right)}^{2}}\\ \;\vdots \\ \hat{d_{8}}=\sqrt{{\left(x+x_{8}-x_{A}\right)}^{2}+{\left(y+y_{8}-y_{A}\right)}^{2}+{\left(z+z_{8}-z_{A}\right)}^{2}} \end{array}\right.$$

where $x_{A}$, $y_{A}$ and $z_{A}$, are the coordinates of the antenna and $x_{i}$, $y_{i}$ and $z_{i}$ are the coordinates of the resonators at the reference position (see Figure 4). The objective is to determine *x* and *y* where $\hat{d_{i}}$ is the estimated distance between antenna and resonator *i* obtained by measurement (as previously described). Classical resolutions are based on Gauss-Newton methods. Unfortunately, as we have seen, the distances estimated using phase measurement are subjected to errors due to phase unwrapping. Each time an error appears during the unwrapping process, a distance of ±λ is added to the distance variation of one resonator. To tackle this problem, we consider, as a first approach, a simpler minimization of the square error:

(5) $$<\hat{x},\hat{y}>=\arg\min\sum_{i=1}^{8}{\left(\hat{d_{i}}-d_{ci}\right)}^{2}$$

where $d_{ci}$ is the distance between the antenna and resonator *i* obtained with a candidate solution of coordinates $x_{c},y_{c}$. Minimization is realized iteratively using a random search over the set of all possible *x* and *y* coordinates. The algorithm start by computing the distances $d_{ci}$ obtained between the antenna and an initial position and its corresponding square error with the estimated distances, then, a perturbation (following a gaussian distribution) on *x* and *y* is added on this initial position and the new square error is estimated. If the new position has a smaller square error than the one corresponding to the actual iteration, this new position is then used in the next iteration. In the other case, a new perturbation is added to the actual candidate position. Thus, the algorithm iteratively converges toward the estimated position $\hat{x},\hat{y}$. Also, it is important to notice that this algorithm uses all the resonators of the chipless tag (as we have seen in Figure 1 a minimal number of 3 distances is required to estimate the position of the tag).

**Figure 8:**
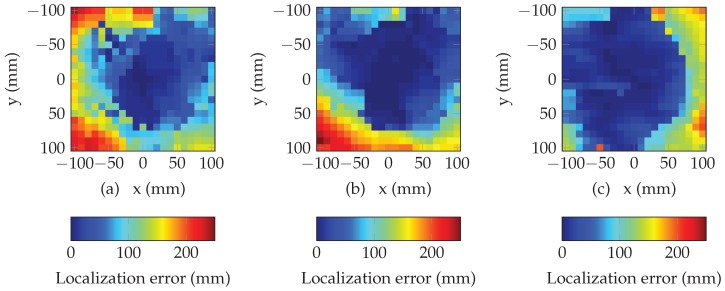
Localization error in mm of the multi-lateration algorithm using 8 resonators for (**a**) d=5 cm, (**b**) d=10 cm and (**c**) d=15 cm.

Figure 8 presents the performance of the multi-lateration algorithm using random search. The plots present the error in magnitude between the estimated position (returned by the algorithm) and the true values x,y of the tag location (known using the positioning table, see Figure 5), for each position. Figure 8a–c plot the results corresponding to heights *h* of 5 cm, 10 cm and 15 cm. For all positions, initial position has been set at x=0 and y=0. The algorithm has been run over 2000 iterations which ensures the convergence (i.e., 10 ms). We can see that multi-lateration algorithm returns an accurate estimation of the location over a large part of the overall space (areas in blue in Figure 8). If we consider a reduced an area of 10 cm centered above the antenna, localization error is lower than 1.98 cm (resp. 1.00 cm and 0.941 cm) for a distance of 5 cm (resp. 10 cm and 15 cm). Moreover, we can easily see that the area where the localization error is low, increases when the distance between the tag and the antenna increases. These results constitute the basic performances of our localization method and will be used as a reference for comparison purpose with the performances with the method presented in the next section.

### 3.3. Selection of a Subset of Resonators

From the results presented in Figure 8, the accuracy of the proposed method is good in an area located above the antenna. However, as the distance between the tag position and the reference position increases, the accuracy becomes lower due to the phase unwrapping error which add (or subtract) a quantity equal to one wavelength in case of measurement noise for some resonators. To improve the performances of our solution, we propose to select a subset of resonators to exclude the ones subjected to phase unwrapping errors (a minimum of three resonators is required). Since these resonators could not be identified during the optimization, we propose to estimate the mean square error for each combination of resonators. Minimization is realized considering all the possible combinations. Note that the number of combinations increases rapidly a subset of resonator is considered which directly affects the execution time.

**Figure 9:**
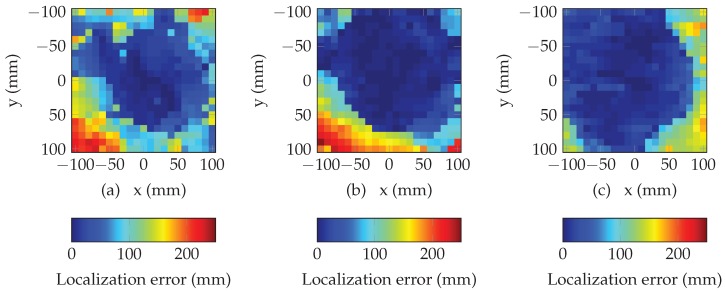
Performance of the multi-lateration algorithm using 7 resonators for (**a**) d=5 cm, (**b**) d=10 cm and (**c**) d=15 cm.

**Figure 10:**
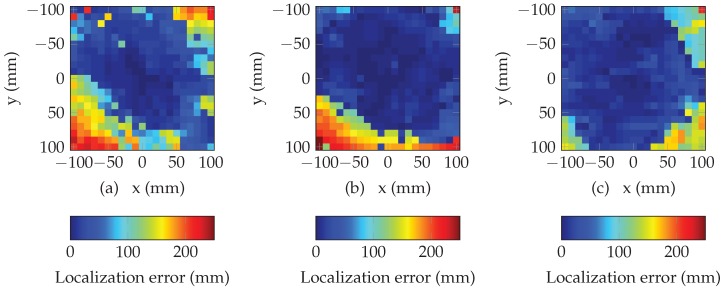
Performance of the multi-lateration algorithm using 6 resonators for (**a**) d=5 cm, (**b**) d=10 cm and (**c**) d=15 cm.

Figure 9 and Figure 10 present the results of the selection of a subset of resonators. We can see that the selection of a subset of different resonators based on the minimization of the mean square error can effectively increase the size of the localization area without reducing the overall accuracy. Moreover, this method permits to increase the performances of the multi-lateration algorithm for all the presented distances. Results are summarized in Table 1 where the proportion of total area where the the error is less than 50 mm, is determined for different subsets and distances. We can see that the proposed method permits to increase the localization area. In average, this area can be increased by a factor higher than 20% by considering a subset of five resonators.

## 4. Conclusions

In this paper we have shown that we can successfully localize a chipless tag using phase measurement over a significant area and at different heights. Distance estimation has been obtained using phase difference between two positions. Accuracy of the proposed method is higher than 2 mm but remains sensitive phase unwrapping errors for displacement longer than one wavelength. Selection of resonators has been proposed to overcome this limitation. Final results show that a localization error less than 1 cm can be achieved in a square of 10 cm side located above the antenna. Finally, this robust localization can be realized without reducing the coding capacity since the same resonators are used for classical reading (based on magnitude) and sensing (based on phase). Finally, this method can easily be extended to determine 3D positioning and/or orientation to provide a wireless, batteryless, printable, possibly disposable and very low-cost positioning system.

## Figures and Tables

**Table 1:** Proportion of the total area (20 cm × 20 cm) where the localization error is less than 50 mm.

	d=5 cm	d=10 cm	d=15 cm
8 resonators	38.8%	57.1%	65.5%
7 resonators	48.8%	68.3%	73.7%
6 resonators	61.7%	78.9%	80.3%
5 resonators	66.4%	84.6%	80.0%
4 resonators	59.6%	78.0%	78.7%
3 resonators	45.6%	63.7%	68.7%
